# Circ_0084043-miR-134-5p axis regulates PCDH9 to suppress melanoma

**DOI:** 10.3389/fonc.2022.891476

**Published:** 2022-10-25

**Authors:** Guiyue Cai, Ruitao Zou, Huizhi yang, Jiahao Xie, Xiaoxuan Chen, Chunchan Zheng, Sujun Luo, Na Wei, Shuang Liu, Rongyi Chen

**Affiliations:** ^1^ Dermatology Department, Dermatology Hospital, Southern Medical University, Guangzhou, China; ^2^ Clinical School, Guangdong Medical University, Zhanjiang, China; ^3^ Dermatology Department, The Third Affiliated Hospital of Guangzhou Medical University, Guangzhou, China

**Keywords:** PCDH9, circRNAs, malignant melanoma, Pyk2, RAC1, apoptosis

## Abstract

The low survival rates, poor responses, and drug resistance of patients with melanoma make it urgent to find new therapeutic targets. This study investigated whether the circ_0084043-miR-134-5p axis regulates the antitumor effect of protocadherin 9 (PCDH9) in melanoma. Ectopic expression or knock down (KD) of PCDH9 with a lentivirus vector, we explored its effects on the proliferation, invasion, and apoptosis of melanoma and verified its regulatory effect on ras-related C3 botulinum toxin substrate 1 (RAC1), proline-rich tyrosine kinase 2 (Pyk2), Cyclin D1, matrix metalloproteinase 2 (MMP2), and MMP9. We further observed the effect of KD circ_0084043 on the malignant behavior of melanoma and studied whether circ_0084043 sponged miR-134-5p and regulated PCDH9. We found that circ_0084043 was overexpressed in melanoma and associated with the malignant phenotype. PCDH9 was poorly expressed in human melanoma tissues, and overexpression of PCDH9 inhibited melanoma progression. Quantitative real-time PCR and Western blotting results showed that overexpression of PCDH9 could downregulate RAC1, MMP2, and MMP9 and upregulate Pyk2 and Cyclin D1. Circ_0084043 KD inhibited invasion and promoted apoptosis in melanoma cells. Circ_0084043 could sponge miR-134-5p and thus indirectly regulate PCDH9. Furthermore, we discovered that inhibiting circ_0084043 had an anti–PD-Ll effect. *In vivo*, PCDH9 overexpression inhibited melanoma tumor growth, but PCDH9 KD promoted it. In conclusion, PCDH9, which is regulated by the circ 0084043-miR-134-5p axis, can suppress malignant biological behavior in melanoma and influence the expression levels of Pyk2, RAC1, Cyclin D1, MMP2, and MMP9.

## Introduction

Malignant melanoma (MM), a type of skin cancer, is not sensitive to radiotherapy and chemotherapy with a tendency for early distant metastases. According to data from the National Cancer Institute, the incidence of melanoma has rocketed over the last 30 years, and even the Cancer Research UK estimated that melanoma has an incidence rate of 3.1/10,000 and a mortality rate of 0.8/10,000 worldwide. Albeit number of validated biomarkers have been confirmed in melanoma, the Chinese population has been demonstrated to gain different subtypes of melanoma from the Caucasian population (1). Therefore, the already-being biomarkers have not been predicted well in the prognosis of Chinese patients with melanoma. About 50% of patients with melanoma carry the v-raf murine sarcoma viral oncogene homolog B1 (BRAF) mutation, which causes the overactivation of MAPK/ERK signaling pathway and leads to the occurrence and metastasis of tumors (2). The combination of BRAF and MAPK ERK kinase (MEK) inhibitors is the main treatment for BRAF mutant melanoma. The curative effect is distinct, but there have been a large number of drug-resistant cases (3). Hence, a novel feasible biomarker is urgently needed.

Bioinformatics technologies have been applied to screen genes that may be relevant to the Chinese population, and the results indicated that PCDH9 was closely related to the prognosis of patients with MM, whereas RAC1 always played the opposite role in our natural bioproduct against melanoma study (4, 5). PCDH9 belongs to protocadherins, one of the largest subfamilies of calcium-dependent adhesion proteins (6). Previous studies have demonstrated that the loss of PCDH9 is associated with the proliferation, differentiation (7–10), and metastasis (7, 10, 11) of tumor cells and predicts poor survival in different cancers (7, 12). RAC subfamily belongs to the RhoGTPase family, including RAC1, RAC2, RAC3, and RhoG. RAC1^P29S^ is the third most common mutation in melanoma after BRAF^V600E^ (50%) and NRAS^Q61^ (20%), accounting for 4% (13). Studies have shown that endogenous RAC1^P29S^ in BRAF^V600E^ mutant melanoma is related to early resistance to BRAF inhibitors, and silencing RAC1^P29S^ expression can restore the sensitivity of cells to BRAF inhibitors (14). RAC1 is reported to regulate cell proliferation, apoptosis, metastasis, and membrane transport by binding and activating guanosine triphosphate (GTP) (15). Our previous study found that RAC1 could recruit CD11b ^+^ Gr1 ^+^ cells and interact with keratin 17, which plays a decisive role in promoting tumorigenesis of skin cancer (5). Inhibition of the RAC1 signaling pathway can reverse the malignant phenotype of squamous cell carcinoma (16). Furthermore, activities of RAC1 have been reported involving different stages of oncogenesis such as initiation, progression, invasion, and metastasis (17), and it was even ranked as the third most frequently occurring mutation in melanoma induced by UV (18, 19).

In addition, it is reported that reactive oxygen species (ROS) are involved in tumor cell migration and invasion (20, 21). ROS was a key component of the nicotinamide adenine dinucleotide phosphate (NAPDH)–oxidase complex formed by RAC1, and RAC1 was one of the major enzymatic sources of ROS in various tissues (22). Reports also emerged that ROS/RAC1–NADPH–oxidase complex is involved in endothelial migration by the mediation of angiotensin-1 and vascular endothelial growth factor (23, 24). As known, endothelial migration is essential for tumor cell invasion, where ROS/RAC1–NADPH–oxidase complex induces expression of matrix metalloproteinases (MMPs) after growth factor and tumor promoter stimulation (25, 26). Tumor-associated MMP family has been studied for over half a century and is known as mediators in some cell processes (27). MMP2 has been believed to act as a pro-tumorigenic and pro-metastatic factor in different cancers aside from melanoma (28), whereas higher MMP9 levels have been found in patients with melanoma than in healthy population, making it a promising marker for melanoma (29). In addition, previous studies have found that MMP9 is activated during the invasion of melanoma cells (29, 30). Proline-rich tyrosine kinase 2 (Pyk2) is a paralog to focal adhesion kinase (FAK) in non-vertebrate species (31) and, in the absence of FAK, can execute the same therapeutic effects (32–34). Increasing Pyk2 and FAK expression could trigger neoplastic activation and promote invasiveness with metastasis of different cancers, highlighting that Pyk2 is a potential therapeutic target for cancer (35, 36). Another key molecule of melanoma is the cell cycle regulator Cyclin D1, which has been demonstrated as an oncogene in cutaneous melanoma (37), and it could affect tumorigenesis *via* nuclear trafficking by multiple mechanisms (38). Therefore, we have chosen MMP2, MMP9, Pyk2, and Cyclin D1 as tumor metastasis indicators to explore the mechanism of how PCDH9 and RAC1 act on melanoma.

Circular RNAs (circRNAs) are a new type of long non-coding RNAs with no 5′ or 3′ polar structure. They widely exist in various eukaryotic organisms and can be used as potential biomarkers of cancer (39). circRNAs are rich in microRNA (miRNA) binding sites and can act as a miRNA sponge to prevent the interaction between miRNA and mRNA in the 3′ untranslated region. Consequently, they indirectly regulate the expression of target genes in this way, playing an important role in the occurrence and development of tumors (40). Luan et al. (41) found that circ_0084043 was the most increased circRNA in primary skin MM by gene chip and that circ_0084043-miR153-3p-snail axis could promote MM invasion. However, the internal relations between circRNAs and miRNA are so complex that the specific regulatory mechanism of the circRNA-miRNA axis in MM remains unclear.

This study identified PCDH9 as a potential tumor suppressor gene in MM. Then, we aimed to explore how PCDH9 regulates the pathogenesis and progress of MM. We found that the expression of RAC1, MMP2, and MMP9 was downregulated, whereas Pyk2 and Cyclin D1 were upregulated in MM cells with overexpressed PCDH9. Moreover, circ_0084043 was discovered to regulate PCDH9 and other tumor-associated proteins through sponging miR-134-5p. Therefore, our research reveals the role of the circ_0084043-miR-134-5p-PCDH9 axis in the pathogenesis and progression of MM.

## Materials and methods

### Chemical and reagents

Dulbecco’s modified Eagle’s medium (DMEM), fetal bovine serum (FBS), and phosphate buffered saline (PBS) (pH = 7.2) were purchased from Gibco (Shanghai, China); ethanol (70%), isopropyl alcohol, and Triton X-100 were bought from Sigma-Aldrich (Shanghai, China); Cell Counting Kit-8 (CCK-8) were bought from Dongren Chemical Technology (Shanghai, China); bicinchonininc acid (BCA) protein assay kit was purchased from Thermo Fisher Scientific (Shanghai, China); antibodies were purchased from Cell Signaling Technology (Shanghai, China) and Abcam (Shanghai, China); the primers [circ_0084043, PCDH9, RAC1, Cyclin D1, Pyk2, FAK, MMP2, MMP9, and glyceraldehyde-3-phosphate dehydrogenase (GAPDH)] were designed and synthesized by Sangon Biotech (Shanghai, China); the small interfering ribonucleic acid (siRNA) for circ_0084043 (si-circ_0084043) was designed and synthesized by Ribo Biotech (Guangzhou, China); the miR-134-5p mimics, miR-134-5p inhibitor, and miRNA negative control (miR-NC) were also purchased from Ribo Biotech; GV358-PCDH9 lentivirus and GV358-siRNA lentivirus were designed by Genechem (Shanghai, China); SYBR^®^ Premix Ex Taq™ II and PrimeScript™ RT reagent Kit with genomic DNA (gDNA) Eraser were bought from Takara Bio Inc. (Beijing, China); ultrapure water was obtained from EPED-20TF (Nanjing, China).

### Cell culture

Cell lines A375, A875, SK-MEL-28, G361 (melanoma cell lines), and PIG1 (normal melanocyte line) were bought from the American Type Culture Collection (MD, USA). They were grown in DMEM supplemented with 10% heat-inactivated FBS as well as penicillin (100 IU/ml) and streptomycin (100 μg/ml). Cells were maintained in a CO_2_ incubator at 37°C under a humidified atmosphere (95% air, 5% CO_2_).

### Overexpression by lentivirus infection

Before the experimental process, cells were divided into three groups: blank group (no lentivirus), control group (lentivirus with empty plasmid), and PCDH9 group (lentivirus with overexpressed PCDH9 plasmid).

Different parameters of lentiviral infections were determined for optimizing the experimental conditions: (i) cell seeding density at 0.5 × 10^6^ cell/ml, (ii) infectious time course at 12 h post-infection (hpi), (iii) observed time at 72 hpi, (iv) lentivirus titer of a control group of 2 × 10^8^ TU/ml, (v) lentivirus titer of a transfected group of 1×10^9^ TU/ml, and (vi) MOI (multiplicity of infection) of 20. Briefly, after seeding cells at an appropriate density in six-well plates (1 × 10^5^ cells per well), they were incubated overnight for 30%–40% adherence. They were then centrifuged for 30 s at a slow speed. After including viruses (MOI = 20), the fresh medium with polybrene was added to maintain 1 ml each, whereas the blank groups contained 1 ml of culture medium with 10% FBS. After being incubated for 12 h, the medium with lentiviruses was changed with a culture medium with 10% FBS (2 ml). Cells were observed by an inverted fluorescent microscope at 72 hpi and were screened by puromycin for post-infection assay.

### KD by lentivirus infection

Before the experimental process, cells were divided into three groups: blank group (no lentivirus), control group (lentivirus with empty plasmid), and PCDH9-siRNA group (lentivirus with siRNA plasmid).

Different parameters of lentiviral infections were determined for optimizing the experimental conditions: (i) cell seeding density at 0.5 × 10^6^ cell/ml, (ii) infectious time course at 12 hpi, (iii) observed time at 72 hpi, (iv) both lentivirus titers of the control group and transfected group of 1 × 10^9^ TU/ml, and (v) MOI was of 20. After seeding cells at the appropriate density in six-well plates (1 × 10^5^ cells per well), they were incubated overnight for 30%–40% adherence. They were then centrifuged for 30 s under slow speed, and after including the viruses (MOI = 20), the fresh medium with polybrene was added to maintain 1 ml each, whereas the blank groups contained 1 ml of culture medium with 10% FBS. After being incubated for 12 h, the medium with lentiviruses was changed with a culture medium with 10% FBS (2 ml). Cells were observed by an inverted fluorescent microscope at 72 hpi and were screened by puromycin for post-infection assay.

### Cell transfection

Before cell transfection, A375 and A875 cells were plated into six-well plates and cultured until 60%–80% confluence, and then, the indicated 10 μM oligonucleotides (si-circ_0084043, si-NC, miR-134-5p mimics, miR-NC, miR-134-5p inhibitor, and miR-inhibitor-NC) were transfected into cells by using Lipofectamine RNAiMAX (Invitrogen) and the cDNA plasmids by using Lipofectamine 3000 (Invitrogen) according to the manufacturer’s protocol.

### Cell viability by Cell Counting Kit-8

Cells were seeded into 96-well plates at a density of 2 × 10^5^ cells per well and treated by a non-transfected plasmid, transfected with the empty plasmid, and transfected with PCDH9 overexpressing plasmid as explained above. After incubation at 24, 48, 72, and 96 h, 10 μl of CCK-8 was added to each well, and cells were incubated for another 4 h at 37°C. The level of colored formazan derivative was analyzed on Thermo Scientific Multiskan FC (Vantaa, Finland) at a wavelength of 450 nm. The viable cells were directly proportional to the formazan production, and the percentage of viable ones was calculated.

### Apoptosis detection by flow cytometer

Apoptosis was measured using the flow apoptosis assay. Cells were seeded in a six-well plate at a density of 1 × 10^6^ cells per well and treated as explained above. At the end of the treatment, cells were centrifuged (1,000 rpm for 10 min). After that, the cells were washed twice by cold PBS and resuspended in 1 ml of 1× Binding Buffer; to 100 μl of this solution, 5 μl of FluoresceinIsothiocyanate (FITC) Annexin V (BD, USA) and 5 μl of Propidium Iodide (PI) (BD, USA) were added. After being vortexed gently and incubated in the dark at room temperature for another 15 min, 400 μl of 1× Binding Buffer was added to the final solution. Finally, samples were analyzed using the BD FACSCanto II flow cytometer. On the basis of the FITC Annexin V staining procedure, cells can be divided into apoptosis and non-apoptosis groups.

### Cell cycle assay by flow cytometer

The cell cycle was measured using the flow apoptosis assay. Cells were seeded in six-well plates at a density of 1 × 10^6^ cells per well and treated as explained above. At the end of the treatment, cells were centrifuged (1,000 rpm for 10 min) and then vortexed with 5 ml of cold 75% ethanol; cells were incubated at −20°C for 2 h, washed twice to remove ethanol (first washed in PBS and then washed in Stain Buffer), and centrifuged again (1,000 rpm for 10 min). The cells were resuspended in 0.5 ml of PI/RNase Staining Buffer (BD, USA), and after 15 min of incubation at room temperature, samples were analyzed using the BD FACSCanto II flow cytometer.

### Cell migration assay

Cells were seeded into six-well plates at a density of 1.5 × 10^5^ cells per well and were incubated under cell culture conditions for 24 h until confluent condition. The scratched wounds were created by a sterile 10-μl pipette tip. The suspension cells were washed twice with PBS, and then, DMEM (2% FBS) was added for incubation. The wounds were viewed using an inverted microscope DMI3000B (Leica, Germany) at 0, 24, and 48 h. ImageJ (Maryland, USA) was applied to measure the wound space.

### TRIzol RNA isolation and purity determination

Cells were rinsed by cold PBS and lysed by trypsin, after which they were thoroughly vortexed and centrifuged for 5 min (1,000 rpm), and the supernatant was discarded and refreshed with cold PBS; after spinning for 5 min (1,000 rpm), TRIzol was respectively added, and after 5 min of incubation at room temperature, chloroform was added (0.2 ml of chloroform to 1 ml of TRIzol) according to the amount of TRIzol; after being vortexed vigorously (15 s) and after 3 min of incubation at room temperature, the sample was spun at 4°C for 15 min (12,000×*g*); aqueous phase was taken. RNA was precipitated by isopropyl alcohol (0.5 ml of isopropyl alcohol to 1 ml of TRIzol in homogenized step) after 10 min of incubation at room temperature. The supernatant was discarded, and then, the RNA, which was at the bottom of the tube, was washed with 75% ethanol (1 ml of ethanol to 1 ml of TRIzol in homogenized step). The sample was centrifuged at 4°C for 5 min (7,500×*g*) several times until all ethanol was removed. After being dried for 8 min, the RNA sample was dissolved in diethyl pyrocarbonate (DEPC)-treated water by pipette tip (1 μl of RNA to 39 μl of DEPC-treated water, 1:40 dilution). The A_260_/A_280_ of RNA extracted was above 1.8.

### Reverse transcription and quantitative real-time PCR

The reverse transcription of the prepared RNA sample was performed by MasterCycler Gradient PCR (Thermo Fisher Scientific, USA) using a PrimeScript RT reagent kit (TaKaRa, Japan) according to the SYBR Green qPCR assay introduction. Quantification of genes was performed with the 2^−ΔΔCT^ method, as described previously (42): The sample was cycled (95°C for 5 s and 60°C for 30 s) 40 times by ABI7500Fast Real-time PCR System Amplifier (Thermo Fisher Scientific, USA). The primers designed for genes (circ_0084043, PCDH9, GAPDH, Pyk2, Cyclin D1, MMP2, MMP9, RAC1, and FAK), and their amplicon sizes are shown in **Supplementary Table S1**.

### Western blot analysis

After treatments, cells were lysed in a buffer containing 20 mM Tris-HCl (pH = 7.5), 0.9% NaCl, 0.2% Triton X-100, and 1% protease inhibitor cocktail. The analyses were conducted on cells between the third and the sixth passages.

Total protein concentration was detected by the BCA protein assay kit. Equal amounts of protein (30 μg) of each sample were loaded and separated in a 10% acrylamide sodium dodecyl sulfate-polyacrylamide gel electrophoresis and transferred to immobilon poly(vinylidene fluoride) (PVDF) membranes. Membranes were washed three times with Tris-HCl–buffered saline containing 5% Tween 20 (TBST), blocked with a 5% non-fat dry blocker, and incubated with the primary antibodies at 4°C overnight. Next, membranes were washed three times with TBST and then incubated with the secondary antibodies. Protein bands were visualized using Immobilon Western Chemiluminescent Substrate, and the protein signals were detected by Azure c500 Infrared Western Blot Imaging System from Azure Biosystems (CA, USA). Quantification of protein expression was made using the software ImageJ (Maryland, USA) and Origin 2020 for visualization (Northampton, MA, USA).

### Immunohistochemistry stains

Immunohistochemistry (IHC) was performed on collected tissues. Forty-five normal human skin tissues, 30 humans pigmented nevus tissues, and 30 human MM tissues were selected. These tissues were ethically acquired from the outpatient clinic of the Affiliated Hospital of Guangdong Medical University, and all of them are Chinese population (Han people) with personal identifiers redacted. These MM tissues were from patients diagnosed with MM by skin pathology from 2012 to 2019, and the pigmented nevus tissues came from patients who underwent surgical resection in dermatologic surgery at the same time. The age, sex, site of onset, Breslow thickness, ulcer condition, and clinical stage of patients with MM included in the sample are shown in **Table 1**. All the tissues were prepared as paraffin specimens. Deparaffinization included two 100% xylene changes (xylene I for 10 min and xylene II for 10 min) followed by rehydration with a graded series of ethanol (anhydrous ethanol I for 5 min; anhydrous ethanol II for 5 min; 95%, 85%, and 75% ethanol all for 5 min) and then rinsed under distilled running water for 3–5 min. Antigen retrieval consisted of 2 min of incubation of slides in citric acid retrieval solution heated to 98°C with a commercial steamer following a cool down step to room temperature (cold water and ice pack were added); slides were transferred into a wet box and were then rinsed three times with PBS. After protein blocking, primary antibodies (1:200) were incubated at 4°C overnight. After being at room temperature for 30 min, the slides were washed three times for 3 min each with PBS. After removing PBS and protein blocking, secondary antibodies (1:1000) were added at room temperature for 1 h. The slides were then washed three times for 3 min each with PBS. After removing PBS, 1 drop of prepared diaminobenzidine (DAB) solution (1 ml of A:1 drop of B:1 drop of C) for DAB staining was added, and the slides were observed under a microscope. After being rinsed in running water for 10 min, hematoxylin was added for 1 min, and then, the slides were washed with water for 5 min. The slides were then dehydrated in a series of ethanol (75%, 85%, 95%, and 100%) and 100% xylene changes and mounted with a coverslip with dry neutral resin.

**Table 1:** The age, sex, site of onset, Breslow thickness, ulcer condition, and clinical stage of MM sample.

Index	Category	Total
Sex	Male	16
	Female	14
Age	≤50	6
	>50	24
Site	Acral	15
	Non-acral	15
Breslow thickness	≤2 mm	12
	>2 mm	18
Ulcers	Existent	11
	Non-existent	19
Clinical stages	I, II	13
	III, IV	17

### Evaluation of various protein expressions in MM

Evaluation of various protein expressions in MM was evaluated by semi-quantitative analysis, according to the staining intensity and the percentage of positive cells. The score standards of staining intensity were as follows: no coloration, 0; low intensity (light yellow), 1; medium intensity (light brown), 2; and high intensity (dark brown), 3. Five fields of view were randomly selected under a microscope (400×), and 500 cells were counted as one unit; meanwhile, the percentage of positive cells was calculated. The percentage scores were as follows: <5%, 0; 6%~25%, 1; 26%~50%, 2; 51%~75%, 3; and >75%, 4. The score standards were the product of staining intensity and percentage of positive cells: 0, negative (−); 1 to 4, positive (+); 5 to 8, moderately positive (++); and 9 to 12, strongly positive (+++).

### Small RNA sequencing

A375 cell was transfected with si-circ_0084043 and si-NC. After 48 h, the total RNA was extracted with TRIzol reagent. An amount of 3 µg of total RNA per sample was used as input material for the small RNA library. Following the manufacturer’s recommendations, sequencing libraries were generated using NEBNext^®^Multiplex Small RNA Library Prep Set for Illumina^®^ (NEB, USA), and index codes were added to attribute sequences to each sample. Briefly, NEB 3′ SR Adaptor was directly and specifically ligated to the 3′ end of miRNA, siRNA, and piwi-interacting RNA (piRNA). After the 3′ ligation reaction, the SR RT Primer hybridized to the excess of 3′ SR Adaptor (that remained free after the 3’ ligation reaction) and transformed the single-stranded DNA adaptor into a double-stranded DNA molecule. This step is important to prevent adaptor-dimer formation. Moreover, double-stranded RNAs (dsRNAs) are not substrates for ligation-mediated by T4 RNA Ligase 1 and therefore do not ligate to the 5′ SR Adaptor in the subsequent ligation step. 5′-End adapter was ligated to 5′ ends of miRNAs, siRNA, and piRNA. Then, first-strand complementary DNA (cDNA) was synthesized using M-MuLV Reverse Transcriptase (RNase H^-^). PCR amplification was performed using LongAmp Taq 2X Master Mix, SR Primer for Illumina, and index (X) primer. PCR products were purified on an 8% polyacrylamide gel (100 V, 80 min). DNA fragments corresponding to 140–160 bp (the length of small non-coding RNA plus the 3′ and 5′ adaptors) were recovered and dissolved in an 8-μl elution buffer. At last, library quality was assessed on the Agilent Bioanalyzer 2100 system using DNA High Sensitivity Chips.

According to the manufacturer’s instructions, the clustering of the index-coded samples was performed on a cBot Cluster Generation System using TruSeq SR Cluster Kit v3-cBot-HS (Illumia). After cluster generation, the library preparations were sequenced on an Illumina Hiseq 2,500/2,000 platform, and 50-bp single-end reads were generated.

### Dual-luciferase reporter assay

The wild-type and mutant fragments of circ_0084043 were inserted into pSI-Check2 vectors. A375 and A875 cells were plated into 24-well plates and cultured at 37°C overnight. Then, the cells were cotransfected with the luciferase reporter vector and miR-134-5p mimic or miR-134-5p inhibitor using Lipofectamine 3000 (Invitrogen). After 48 h of transfection, luciferase activities were detected by the Dual-Luciferase Reporter Assay System (Promega, WI, USA).

### Protein complex immunoprecipitation

PCDH9-Flag was overexpressed in A375 and G361 cell lines by lentivirus infection and cultured by DMEM with 10% FBS in 10-cm petri dishes as mentioned above. After discarding the cell medium and washing once by iced PBS, the cells were lysed in a buffer containing TBS with 1% Triton X-100 and phenylmethanesulfonyl fluoride (PMSF), 1 ml in each dish. The solution was collected in 1.5-ml Eppendorf tubes, and after centrifugation (1,000 rpm for 15 min), the supernatant was collected. For the input sample preparation, 80 μl of protein solution with 20 μl of 5× loading buffer was boiled. The leftover solution was incubated with 2 μl of anti-Flag antibody at 4°C overnight. Protein G Agarose beads (40 μl each tube) were added, and the solution was shaken for 3 h at 4°C. Then, after being centrifuged (2,500 rpm for 5 min), the supernatant was removed. The sediments were washed five times by lysis buffer with PMSF (1 ml each time) and were boiled for 10 min at 100°C after adding loading buffer (40 μl). The control group was incubated with mouse immunoglobulin G (IgG).

### Tumor growth assay *in vivo*

All animal experiments were subject to approval by the Institutional Review Board of Guangdong Medical University (Zhanjiang, China). Six-week-old nude female mice were kept in the specific pathogen free (SPF) animal room. A375 and A375 cells (5 × 10^6^) transfected with PCDH9 overexpressing plasmid, PCDH9 KD plasmid, or empty plasmid in 150 μl of PBS were inoculated subcutaneously in the mice. After 35 days, the mice were sacrificed to remove the tumors, and the tumor size of each group was measured.

### Statistical analysis

Data are presented as the mean ± SD from at least three independent experiments. Statistical comparisons between two groups were made using Student’s *t*-tests. Differences among three or more groups were compared by one-way ANOVA, followed by least significant difference *post-hoc* tests (Originlab 2020, Northampton, MA, USA); *p* < 0.05 was considered statistically significant, whereas *p* < 0.01 was considered highly statistically significant.

## Results

### PCDH9 expression affected selected genes expression and proteins expression

**Figure 1:**
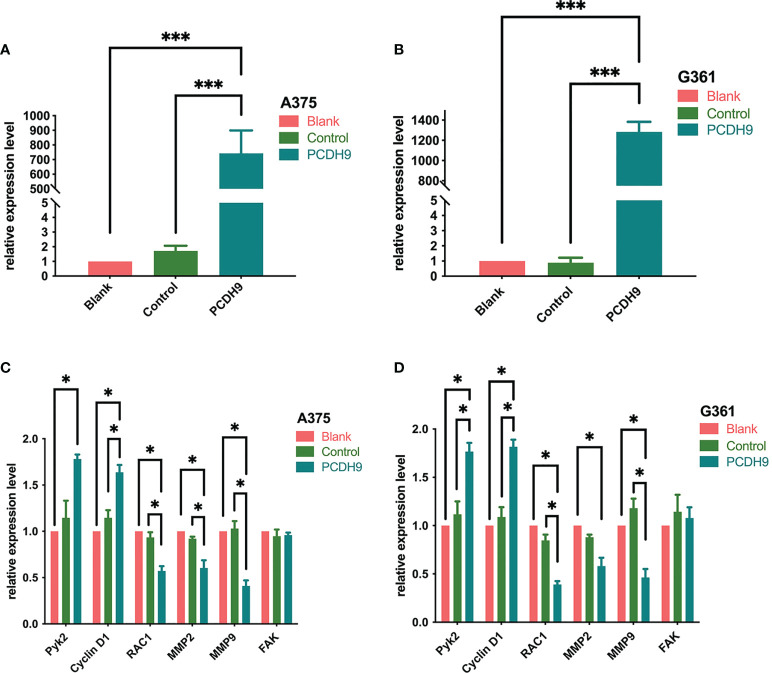
Effects of PCDH9 overexpression in melanoma cells measured by qRT-PCR. The expressions of PCDH9 were significantly upregulated by lentivirus infection **(A, B)**. Overexpressed PCDH9 significantly upregulated the gene expressions of Pyk2 and Cyclin D1 and downregulated RAC1, MMP2, and MMP9 except for FAK in A375 **(C)** and G361 **(D)** cells. *p < 0.05 and ***p < 0.001 compared groups using one-way ANOVA followed by least significant difference *post-hoc* tests. The results cited from the preprint doi: https://doi.org/10.21203/rs.3.rs-95026/v1.

**Figure 2:**
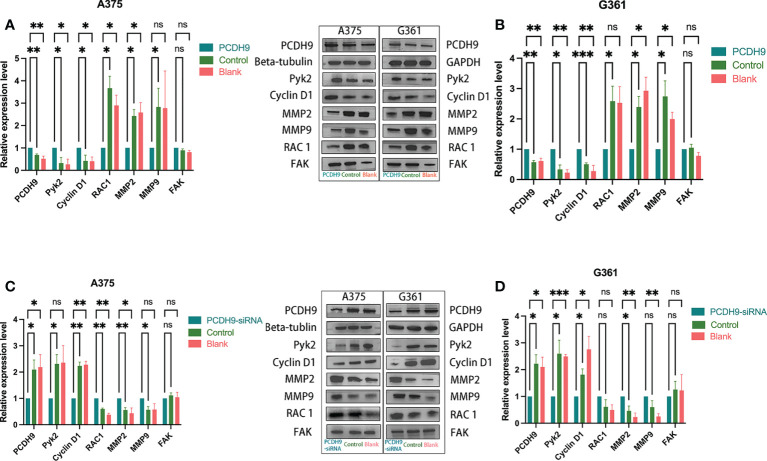
Effects of PCDH9 overexpression and KD in melanoma cells measured by Western blot. Overexpressing PCDH9 significantly upregulated levels of Pyk2 and Cyclin D1 and downregulated RAC1, MMP2, and MMP9 in A375 **(A)** and G361 **(B)** cells. KD of PCDH9 downregulated levels of Pyk2 and Cyclin D1 and upregulated RAC1, MMP2, and MMP9 in A375 **(C)** and G361 **(D)** cells. ^ns^ *P* > 0.05, *p < 0.05, **p < 0.01, and ***p < 0.001 compared groups using one-way ANOVA followed by least significant difference *post-hoc* tests.

PCDH9 was overexpressed by lentivirus with PCDH9 plasmid (**Figures 1A, B**) and knocked down by lentivirus with siRNA (**Figures 2C, D**). The relative expression of selected genes (Pyk2, Cyclin D1, RAC1, MMP2, and MMP9 except for FAK) varied with PCDH9 expression, but their effectiveness differed. Pyk2, Cyclin D1, and PCDH9 exhibited a positive correlation, whereas RAC1, MMP2, MMP9, and PCDH9 negatively correlated in both A375 and G361 cells (**Figures 1C, D**). The results of quantitative real-time PCR (qRT-PCR) were confirmed by Western blotting analysis. Except for FAK, the protein expressions of Pyk2, Cyclin D1, MMP2, MMP9, and RAC1 had a significant correlation with PCDH9 in A375 and G361 cells (**Figure 2**). PCDH9 and Pyk2/Cyclin D1 exhibited a positive correlation, whereas PCDH9 and RAC1/MMP2/MMP9 negatively correlated (**Figure 2**). Therefore, the overexpression of PCDH9 upregulated the gene expressions of Pyk2 and Cyclin D1 and downregulated RAC1, MMP2, and MMP9, whereas KD of PCDH9 downregulated expression levels of Pyk2 and Cyclin D1 and upregulated RAC1, MMP2, and MMP9.

### Effects of overexpressed PCDH9 on cell viability and apoptosis

**Figure 3:**
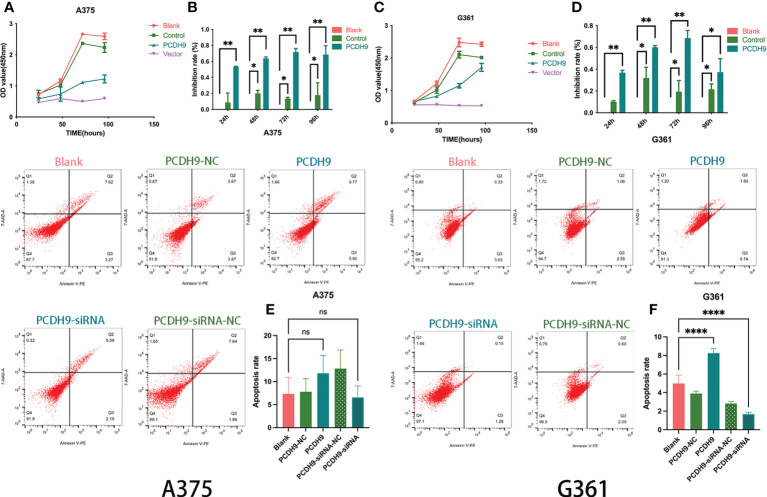
The viability of melanoma cells was significantly reduced by PCDH9 overexpression in A375 **(A, B)** and G361 **(C, D)** cells. *p < 0.05, **p < 0.01, and ***p < 0.001 compared groups using one-way ANOVA followed by least significant difference *post-hoc* tests. The alteration of PCDH9 expression significantly affected the apoptosis of melanoma cells. The overexpression of PCDH9 significantly promoted apoptosis in G361 cells and a more modest manner in A375 cells, whereas PCDH9 KD reduced apoptosis in G361 cells and a more modest manner in A375 cells **(E, F)**. ^ns^ *P* > 0.05, *p < 0.05, **p < 0.01, and ****p < 0.0001 compared with blank group by using Student’s *t*-tests.

The overexpression of PCDH9 reduced the proliferation of melanoma cells. Overexpressed PCDH9 groups showed lower viability than blank/control groups (**Figures 3A, C**). As time passed, the viability of melanoma cells trended to stabilize, but the inhibition rate of cell proliferation in overexpressed PCDH9 groups was higher than that in the blank/control groups, and the differences were significant at 24, 48, 72, and 96 h (**Figures 3B, D**).

The expression of PCDH9 significantly affected the apoptosis of melanoma cells as well. Overexpression increased apoptosis (*p* < 0.05), whereas KD decreased apoptosis (*p* < 0.05) in G361 cells (**Figure 3F**), and a similar phenomenon happened in A375 cells with more modest effects (**Figure 3E**).

Recent studies have found low PCDH9 expression in various cancer types (10, 43). The above phenomenon agreed with our investigation where alterations of PCDH9 could influence the viability of melanoma cells. Meanwhile, the percentage of apoptosis cells was also affected by PCDH9.

### Effects of overexpressed PCDH9 on cell cycle and migration

**Figure 4:**
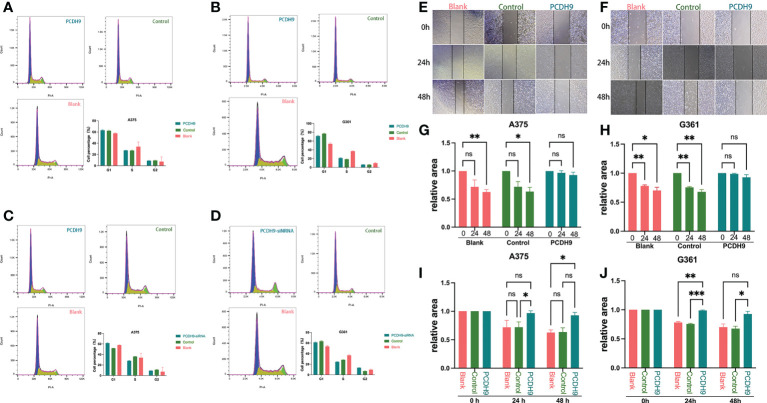
The alteration of PCDH9 expression did not affect the melanoma cell cycle. The cell percentage of A375 **(A)** and G361 **(B)** cells in G1, S, and G2 phases when PCDH9 was overexpressed. The cell percentage of A375 **(C)** and G361 **(D)** cells in G1, S, and G2 phases when PCDH9 interfered. *p < 0.05, **p < 0.01, and ***p < 0.001 compared groups using one-way ANOVA followed by least significant difference *post-hoc* tests. Overexpression of PCDH9 inhibits the migration of melanoma cells. Scratch width of A375 **(E)** and G361 **(F)** cells at 0, 24, and 48 h. The scratch area of A375 **(G)** and G361 **(H)** cells overexpressing PCDH9 did not alter at 24 and 48 h. After 24 and 48 h, the relative scratch area of the PCDH9 overexpression group was larger than that of the blank group and negative control group in A375 **(I)** and G361 **(J)** cells. ^ns^P > 0.05, *p < 0.05, **p < 0.01, ***p < 0.001 compared groups using one-way ANOVA followed by least significant difference post hoc tests.

Regarding the cell cycle arrest, although we found differences between control and KD PCDH9 groups in G361 cells, there was no discrepancy between overexpressed/KD PCDH9 groups and other groups (blank and control groups) in both cell lines (A375 and G361) (**Figures 4A–D**). Therefore, the alteration of PCDH9 did not affect the melanoma cell cycle, and the cell cycle regulator Cyclin D1 modulation revealed no significant effect on the cell cycle. Previous investigations of hepatocellular carcinoma (HCC) found that PCDH9 suppresses HCC cells by inducing cell cycle arrest at G0/G1 phase (8). This result suggests that PCDH9 and Cyclin D1 may affect melanoma cells through other mechanisms.

Concerning cell migration, after quantifying the scratched boundary by ImageJ (**Figures 4E, F**), the results revealed that the scratch area decreased with the duration of cell culture in blank and control groups (*p* < 0.05), whereas the relative wound width did not change markedly in PCDH9 overexpressed groups (*p* > 0.05) (**Figures 4G, H**). The relative wound width of the scratched boundary was significantly different in PCDH9 overexpressed groups compared with blank and control groups after 24 and 48 h (*p* < 0.05) (**Figures 4I, J**). This phenomenon is similar to the correlation between PCDH9 and other tumor cells (HCC) (11, 43). To conclude, overexpressing PCDH9 could significantly inhibit melanoma cell migration by decreasing cell viability and increasing apoptosis.

### Interaction of PCDH9 with RAC1 and Pyk2

The interaction between Pyk2 and other proteins could affect the function of Pyk2 as previously demonstrated (2, 40). In the current study, the expressions of Pyk2 and RAC1 in melanoma cells were significantly affected by the alteration of PCDH9 (**Figures 1B, C**, **2B**). Therefore, we explored the interaction between PCDH9 and Pyk2/RAC1 by complex immunoprecipitation. As a result, PCDH9 and Pyk2/RAC1 were co-immunoprecipitated with PCDH9-FLAG in both cell lines (A375-PCDH9-Flag and G361-PCDH9-Flag), whereas there was no protein in the negative control (**Supplementary Figure S1**). It was demonstrated that PCDH9 interacted with the protein Pyk2 and RAC1. On the basis of previous investigations, we hypothesized that PCDH9 may exert the function of Hace1 through a similar mechanism; however, Hace1 targets complex-bound RAC1 directly; whereas PCDH9 needs the help of Pyk2 (44). The results cited from the preprint https://doi.org/10.21203/rs.3.rs-95026/v1.

### Circ_0084043 was increased in melanoma cells and PCDH9 was decreased in melanoma tissue

**Figure 5:**
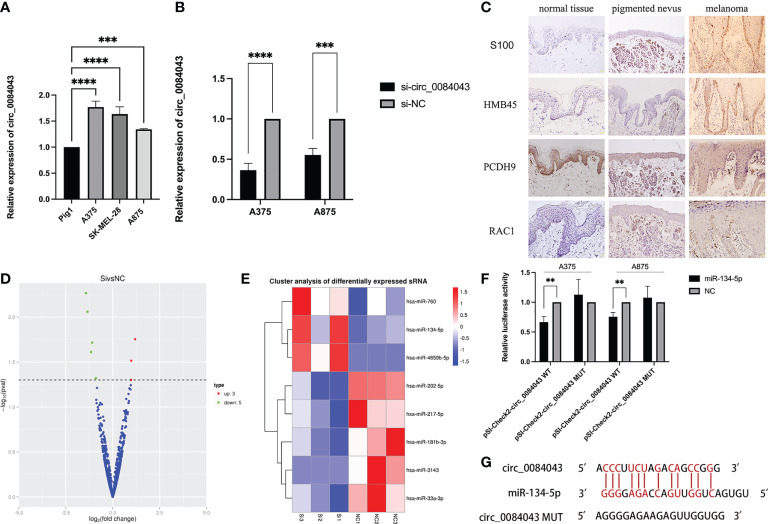
qRT-PCR results showed the expression of circ_0084043 in human melanoma cell lines (A375, SK-MEL-28, and A875) and normal human epidermal melanocytes cell line (PIG1) **(A)**. Circ_0084043 was knocked down in A375 and A875 cells by siRNA **(B)**. Data represent mean ± SD from three independent experiments. **p < 0.01, ***p < 0.001 and ****p < 0.0001 compared with control groups by using Student’s t-tests. Immunohistochemical analyses of PCDH9 and RAC1 expression in normal skin, pigmented nevus, and melanoma tissue. The positive percentage of PCDH9 expression was lower in human melanoma tissue than in normal skin or/and pigmented nevus tissue and the positive percentage of RAC1 expression was higher in human melanoma tissue than normal skin or/and pigmented nevus tissue. PCDH9 is mainly localized in the cytoplasm, with a small amount localized in the nuclei. S100 and HMB45 are melanoma markers. The scale bar represents 200 μm **(C)**. The volcano map of differential miRNA showed that three miRNAs are upregulated and five are downregulated after knocking down circ_0084043 (the red dot indicates upregulation, the green dot indicates downregulation, and the blue dot indicates no significant difference) **(D)**. Differential miRNA cluster analysis showed that miR-134-5p, miR-4659b-5p, and miR-760 were upregulated, and miR-202-5p, miR-33a-3p, miR-181b-3p, miR-217-5p, and miR-3143 were downregulated after knocking down circ_0084043 (*P* < 0.05) (red indicates high expression of miRNA, and blue indicates low expression of miRNA) **(E)**. Dual-luciferase reporter assays revealed that the luciferase activity of circ_0084043 luciferase reporters was reduced by miR-134-5p mimic (*P* < 0.01) **(F)**. Predicted binding sites of circ_0084043 and miR-134-5p **(G)**.

We verified that circ_0084043 was significantly upregulated in melanoma cells. The expression of circ_0084043 was tested in three melanoma cell lines (A375, A875, and SK-MEL-28) through qRT-PCR. The result showed that circ_0084043 was increased in A375, A875, and SK-MEL-28 cells compared to normal human epidermal melanocytes (PIG1) (**Figure 5A**). In addition, si-circ_0084043 could knock down circ_0084043 in melanoma cells (A375 and A875) (**Figure 5B**). Then, IHC results showed that the positive percentage of PCDH9 expression was lower in human melanoma tissue than in normal skin or/and pigmented nevus tissue, and the positive percentage of RAC1 was just the opposite; additionally, PCDH9 was mainly expressed in the cytoplasm, whereas a small amount was expressed in the nuclei (**Figure 5C**). PCDH9 was 100% expressed in normal skin (45/45) or/and pigmented nevus tissue (30/30), whereas only 23.3% (7/30) in melanoma tissue, which was lower than non-tumor tissue (**Table 2**).

**Table 2:** The positive percentage of PCDH9 expression in normal skin, pigmented nevus, and melanoma tissues.

Tissues	-	1+	2+	3+	Positive percentage
Normal skin (n = 45)	0	2	5	38	100.0%
Pigmented nevus (n = 30)	0	3	9	18	100.0%
Melanoma (n = 30)	23	6	1	0	23.3%

### Circ_0084043 worked as a miR-134-5p sponge in melanoma cells

As known, circRNAs work as a sponge of miRNA in tumor cells. To find more targets of circ_0084043, we performed a small RNA sequencing on A375 cells after circ_0084043 was knocked down. Compared with si-NC group, eight miRNAs differentially expressed in si-circ_0084043 group (*P* < 0.05), in which miR-134-5p, miR-4659b-5p, and miR-760 were upregulated and miR-202-5p, miR-33a-3p, miR-181b-3p, miR-217-5p, and miR-3143 were downregulated (**Figures 5D, E**). Previous studies reported that miR-134-5p was significantly downregulated in patients with melanoma compared with healthy control (45). In addition, we verified the upregulation of miR-134-5p in circ_0084043 KD cells by qRT-PCR. Therefore, we speculated that circ_0084043 could sponge miR-134-5p. Luciferase reporter assays were performed to test the hypothesis. The results indicated that miR-134-5p mimics could reduce the luciferase activity of cells transfected with wild-type sequence of circ_0084043 instead of mutant type sequence (**Figure 5F**). The binding site between miR-134-5p and circ_0084043 was predicted using RNAhybrid 2.2 (**Figure 5G**).

### KD of circ_0084043 inhibited melanoma cell migration and promoted apoptosis *via* sponging miR-134-5p

**Figure 6:**
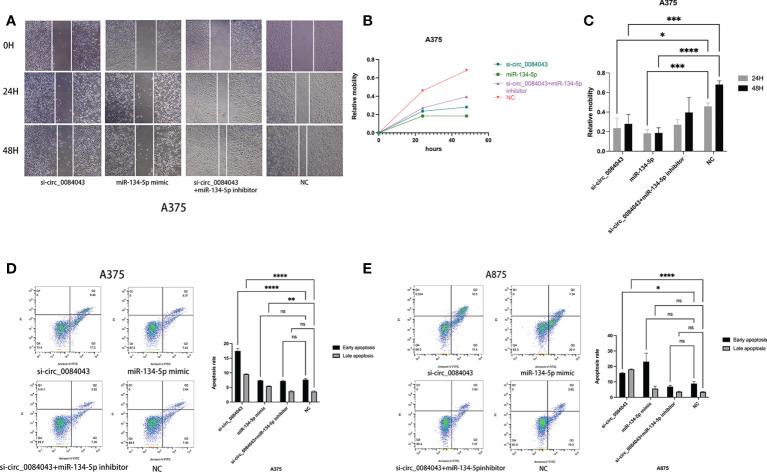
The cell migration ability of A375 cells was detected by scratch wound assay **(A–C)**. Apoptosis was determined by flow cytometry after transfection for 48 h **(D, E)**. Data represent mean ± SD from three independent experiments. *p < 0.05, **p < 0.01, ***p < 0.001, and ****p < 0.0001 compared groups using one-way ANOVA followed by least significant difference *post-hoc* tests.

It is reported that circ_0084043 promoted the growth and metastasis of melanoma cells (41), and KD of circ_0084043 suppressed melanoma progression (46). We further explored the biological role of circ_0084043 and miR-134-5p in the progression of MM. Scratch wound assays showed that the migration abilities of melanoma cells decreased in both the circ_0084043 KD group and miR-134-5p mimic group (**Figures 6A–C**). The flow apoptosis assays demonstrated that the apoptosis of melanoma cells was induced in the circ_0084043 KD group and miR-134-5p mimic group (**Figures 6D, E**). Moreover, the miR-134-5p inhibitor could reverse the effects of inhibiting migration and promoting apoptosis after knocking down circ_0084043 (**Figure 6**). These results indicated that circ_0084043 might promote melanoma progression *in vitro* by sponging miR-134-5p.

### KD of PCDH9 promoted melanoma tumor growth *in vivo*

**Figure 7:**
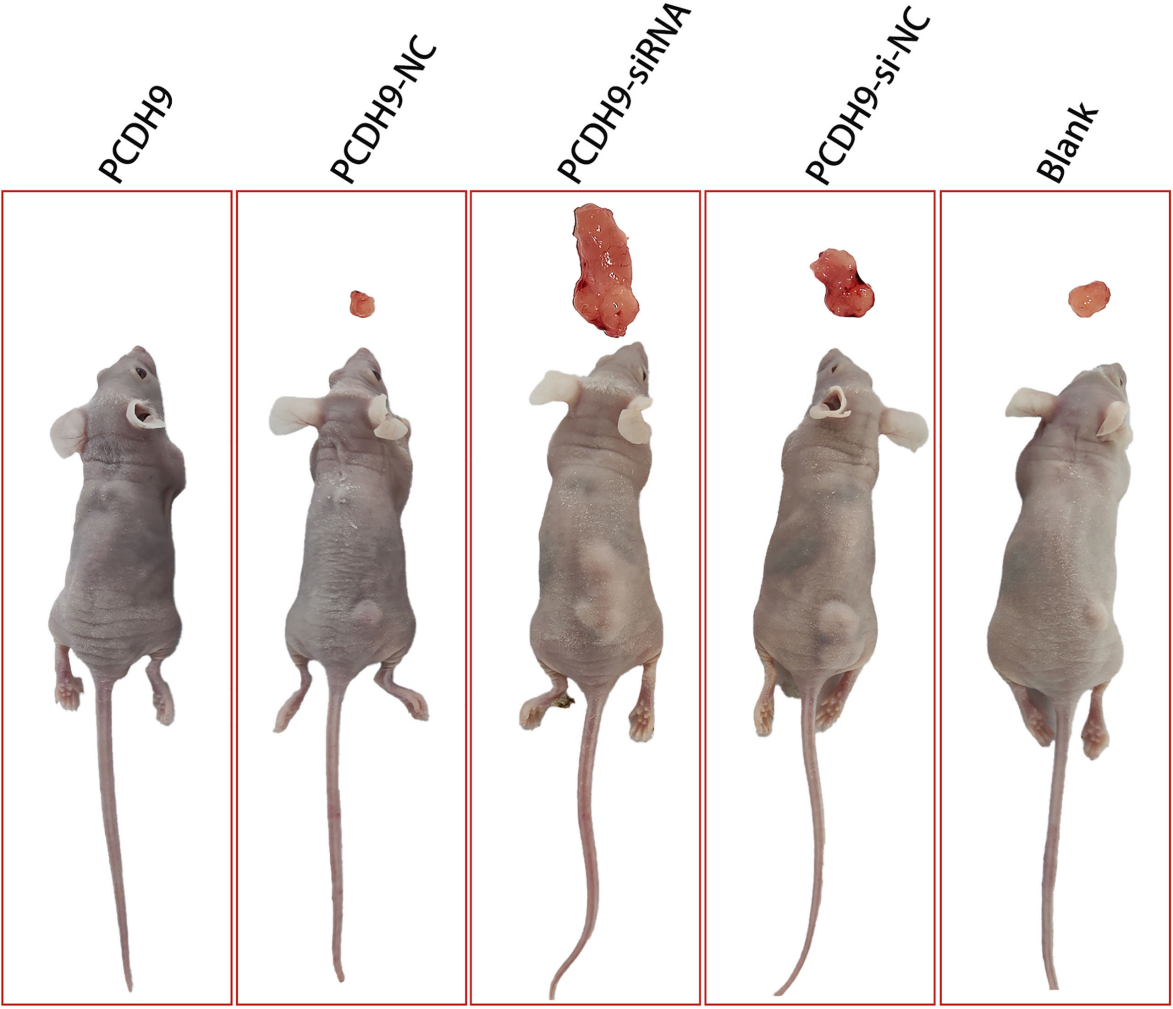
Photographs illustrated tumors in the subcutaneous xenograft tumor model. Tumors in the KD PCDH9 group were significantly heavier than those in the control and blank groups. The nude mice with overexpression of PCDH9 did not grow tumors of significant size.

To further identify the anticarcinogenic of PCDH9 in melanoma, we established the subcutaneous xenograft tumor model using nude mice. We found that tumors in the PCDH9 KD group were significantly heavier than those in the control and blank group (**Figure 7**). The nude mice with overexpression of PCDH9 did not grow tumors of significant size (**Figure 7**). Therefore, animal experiments presented that PCDH9 could work as a tumor suppressor gene to inhibit the growth of MM.

### Circ_0084043 regulated PCDH9 and the expression of the selected genes indirectly in melanoma cells

**Figure 8:**
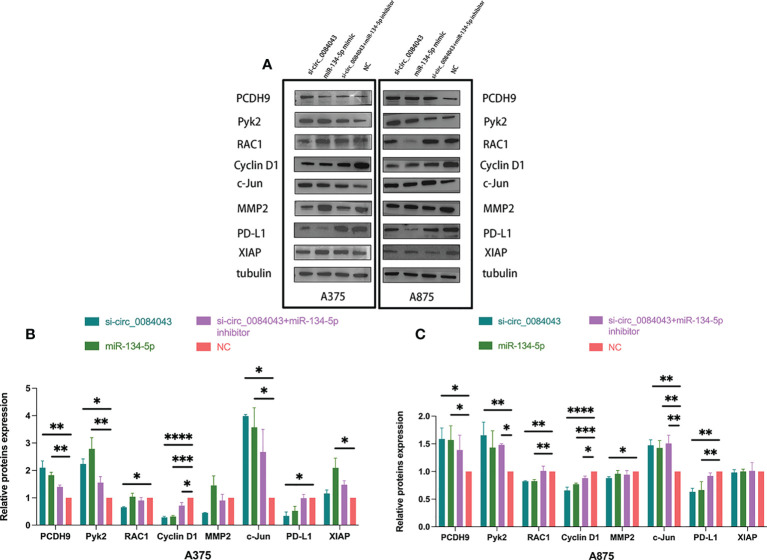
Western blot was used to detect the expression levels of PCDH9, Pyk2, RAC1, Cyclin D1, MMP2, c-Jun, XIAP, and PD-L1 in melanoma cells after transfection for 48 h **(A–C)**. Data represent mean ± SD from three independent experiments. *p < 0.05, **p < 0.01, ***p < 0.001, and ****p < 0.0001 compared groups using one-way ANOVA followed by least significant difference *post-hoc* tests.

The findings indicated that PCDH9 functioned as a tumor suppressor gene in melanoma development, and circ_0084043 is proved to be an oncogene. What is the mechanism of the circ_0084043-miR-134-5p axis promoting cancer in melanoma? We obtained the prediction results of the miR-134-5p target on the TargetScan and Starbase 3.0 database. Hence, we selected the following proteins for research based on the two databases: Kyoto Encyclopedia of Genes and Genomes (KEGG) and Gene Ontology (GO) analysis (**Supplementary Figures S2A, B**). Then, Western blot was carried out to search for the downstream mRNA targeted by the circ_0084043-miR-134-5p axis. As shown in **Figure 8**, the levels of PCDH9, Pyk2, and c-Jun were increased after knocking down circ_0084043 in A375 and A875 cells, whereas RAC1, Cyclin D1, MMP2, and PD-L1 were downregulated. Overexpression of miR-134-5p had the same effect as KD of circ_0084043 except for MMP2. Cotransfection of si-circ_0084043 and miR-134-5p inhibitor could reverse the transfection of circ_0084043 alone except for MMP2. X-linked inhibitor of apoptosis protein (XIAP) is an inhibitor of apoptosis protein highly expressed in melanoma, and we found that its expression level was not affected by circ_0084043 and miR-134-5p. To sum up, the circ_0084043-miR-134-5p axis could regulate PCDH9 and the above tumor-associated proteins in melanoma cells.

## Discussion

BRAF mutation is the most common gene mutation in patients with melanoma. The single-drug treatment of BRAF inhibitor or combined with MEK inhibitor has achieved remarkable curative effect, but a large number of drug-resistant cases have been found, and serious drug-related adverse reactions have occurred in the later stage of treatment (2, 3). Therefore, it is imperative to comprehensively understand the molecular mechanism of melanoma and develop more appropriate therapeutic strategies. Here, we utilized melanoma cell lines with BRAF mutant to study, in the hope of developing new target inhibitors to reduce the occurrence of drug resistance.

With the rapid development of high-throughput sequencing in recent years, a growing number of altered genes and signaling pathways were reported to act in melanoma (47). We found that PCDH9 was closely related to the prognosis of patients with melanoma, and it was lowly expressed in human MM tissues. We delved into the biological function of PCDH9 in melanoma and found that it played the role of the tumor suppressor gene. Alteration of PCDH9 could affect the proliferation, invasion, and apoptosis of MM cells by regulating the expression of Pyk2, Cyclin D1, RAC1, MMP2, and MMP9. *In vivo*, overexpression of PCDH9 inhibited melanoma tumor growth, but KD of PCDH9 promoted it. Wang et al. found that PCDH9 also acted as a tumor suppressor gene in glioma cells to promote apoptosis and overexpression of PCDH9 could upregulate Bax and downregulate Bcl-2 protein levels (48). Studies showed that activated RAC1 could stabilize the anti-apoptotic activity of Bcl-2 through the promotion of the sustained phosphorylation at serine-70 (s70pBcl-2) (49) but the inhibition of RAC1 signaling could promote caspase- induced apoptosis (including caspase-8, caspase-9, caspase-7, and caspase-3) (50, 51). The above evidence indicates that overexpression of PCDH9 may induce apoptosis of tumor cells by regulating apoptosis-related genes (such as Bax, Bcl-2, and caspase). The expression of PCDH9 in various tumor tissues was significantly lower than that in healthy tissues, such as prostate, gastric, HCC, non-nodal mantle cell lymphoma, glioma, and nodal/hepatic metastatic tissues (7, 11, 52). All the findings allow us to recognize that PCDH9 may be a key molecule in the malignant transformation of the melanocyte and the progression of melanoma.

CircRNAs have become one of the hotspots in tumor study, but few reports on melanoma exist. Circ_0084043 was reported to be the most increased circRNA and function as an oncogene to stimulate cell proliferation and metastasis in melanoma (41, 46). Consistently, another report also indicated that circ_0084043 was enhanced in melanoma tissues and cells and circ_0084043 knockdown inhibited cell proliferation, induced cell apoptosis, and repressed glycolysis as well (53). We also discovered that the expression of circ_0084043 was elevated in melanoma cells. Moreover, KD of circ_0084043 suppressed cell migration and induced cell apoptosis by sponging miR-134-5p. MiR-134-5p was significantly upregulated in glioma cells, and the overexpression of miR-134-5p inhibited the proliferation and migration of glioma cells (54). Another study has found that miR-134-5p is significantly downregulated in patients with melanoma (45), but its specific function has not been further studied. Through Western blot assay, we found that circ_0084043 downregulated PCDH9, Pyk2, and c-Jun and upregulated RAC1, Cyclin D1, MMP2, and PD-L1 by sponging miR-134-5p. However, the target of miR-134-5p has not been verified by the luciferase reporting system, RNA binding protein immunoprecipitation, or PCR array using the target gene. The above experiments need further research by our research group.

The expression of MMP2 is a prognostic marker for patients with MM that could independently predict patient survival (55), and its expression has been related to the poorer survival of patients with melanoma (55), consistent with varied PCDH9 expression in melanoma cells. MMP9 could block the EGFR-ERK/AKT signaling pathway to suppress the angiogenesis of MM, and it has been considered a key angiogenic factor (56). The results of MMP9 expression in this study exhibited a negative correlation with PCDH9 and a positive correlation with RAC1. According to previous studies, the roles of Cyclin D1 in melanoma cells are debatable. Some studies suggested that the upregulated expression of Cyclin D1 enhances metastases (57), whereas others found that Cyclin D1 was downregulated in metastatic tumors (58). Even some histopathological studies showed no significant difference in the expression of Cyclin D1 (59). In the present study, we found that Cyclin D1 increased along with the overexpression of PCDH9, which is negatively correlated with MMP2 and MMP9. A previous study showed that Hace1 targeted complex-bound RAC1 to regulate Cyclin D1 expression and ROS generation with ROS-induced DNA damage (44). High DNA damage tends to carcinogenesis. Meanwhile, the previous exploration of how Cyclin D1 affects tumorigenesis speculated that it could play a role in nuclear trafficking rather than overexpressing itself (38, 44). PCDH9 might be trafficked from nuclei to the cytoplasm with the assistance of Cyclin D1.

C-Jun, the major member of the activator protein 1 (AP-1) transcription factor family, was involved in the tumorigenesis, proliferation, invasion, metastasis, and apoptosis of melanoma (60–62). More than one regulatory mechanism could activate c-Jun to promote tumor progression. The c-Jun N-terminal kinase (JNK), with its members JNK1, JNK2, and JNK3, is a subfamily of MAPK. Upon activation, JNK signaling can phosphorylate AP-1 family proteins (63). AP-1 has been proved to regulate the expression of many cell cycle regulators such as p53, p21, p16Ink4a, p19ARF, Cyclin D1, Cyclin A, and Cyclin E (64, 65). Recently, there was an evidence that miR-125b is directly bound to the coding region of c-Jun and affected its downstream molecules (66). Contrary to most studies, Quercetin (a small-molecule compound) can promote tumor cell apoptosis by upregulating c-Jun *in vivo* and *in vitro* (67), and the inhibitor of c-Jun could attenuate this effect (68). Our study found that c-Jun decreased with the KD of circ_0084043 but increased with the cotransfection of si-circ_0084043 and miR-134-5p inhibitor. c-Jun has been considered as a protein promoting tumor progression in most reports previously, but our experimental results did not seem to support this view. What is the relation between c-Jun and PCDH9 and what role does c-Jun play in MM? These uncertainties also need to be further explored by our research group.

PD-L1 is the ligand of PD-1 expressed on the surface of T cells. The combination between PD-L1 and PD-1 will prevent the immune system from recognizing and killing tumor cells. At present, PD-1 monoclonal antibody has been widely used in the clinical treatment of many tumors such as melanoma (69), non–small cell lung cancer (70, 71), and urothelial carcinoma (72, 73). The latest research shows that the combination of targeted therapy and immunotherapy can strengthen the control of tumor growth, but it is accompanied by obvious adverse reactions, such as liver dysfunction and heat radiation disease (74). Our results show that the PD-L1 level is regulated by the circ_0084043-miR-134-5p axis, suggesting that targeting circ_0084043 may contribute to the immunotherapy of MM. It is necessary to study the immune-related mechanisms and signal pathways if we want to verify this hypothesis.

Together, this study found that circ_0084043 regulated PCDH9 and other key molecules, promoted migration, and inhibited apoptosis of MM cells by sponging miR-134-5p. PCDH9 plays an important role in the pathogenesis of MM, and its comprehensive studies are the experimental foundation for the clinical transformation of this target.

## Data availability statement

The data presented in the study is deposited in the NCBI repository, accession number PRJNA888386 (https://www.ncbi.nlm.nih.gov/search/all/?term=PRJNA888386).

## Ethics statement

The animal study was reviewed and approved by The Ethics Committee of Guangdong Medical University (Zhanjiang, China).

## Author contributions

All authors contributed to the study’s conception and design. Material preparation, data collection, and analysis were performed by GC, RZ, and HY. The first draft of the manuscript was written by GC, and all authors commented on previous versions of the manuscript. All authors read and approved the final manuscript.

## Funding

This work was supported by the Natural science foundation of Guangdong Province of China (grant numbers 2021A1515011218 and 2020A1515010281).

## Conflict of interest

The authors declare that the research was conducted in the absence of any commercial or financial relationships that could be construed as a potential conflict of interest.

## Publisher’s note

All claims expressed in this article are solely those of the authors and do not necessarily represent those of their affiliated organizations, or those of the publisher, the editors and the reviewers. Any product that may be evaluated in this article, or claim that may be made by its manufacturer, is not guaranteed or endorsed by the publisher.

## References

[1] Ugurel S Utikal J Becker JC. Tumor biomarkers in melanoma. Cancer control (2009) 16:219–24. doi: 10.1177/107327480901600303 19556961

[2] Gerosa L Chidley C Fröhlich F Sanchez G Lim SK Muhlich J. Receptor-driven ERK pulses reconfigure MAPK signaling and enable persistence of drug-adapted BRAF-mutant melanoma cells. Cell Syst (2020) 11:478–494.e9. doi: 10.1016/j.cels.2020.10.002 33113355 PMC8009031

[3] Doepner M Lee IY Ridky TW. Drug-resistant melanoma may be vulnerable to inhibitors of serine synthesis. J Invest Dermatol (2020) 140:2114–6. doi: 10.1016/j.jid.2020.05.103 33099398

[4] Chen R Zhang G Zhou Y Li N Lin J. A time course-dependent metastatic gene expression signature predicts outcome in human metastatic melanomas. Diagn Pathol (2014) 9:155. doi: 10.1186/s13000-014-0155-2 25116415 PMC4149277

[5] Chen R Fu M Zhang G Zhou Y Zhu S Liu J. Rac1 regulates skin tumors by regulation of keratin 17 through recruitment and interaction with CD11b+Gr1+ cells. Oncotarget (2014) 5:4406–17. doi: 10.18632/oncotarget.2030 PMC4147333 24962779

[6] Kim SY Yasuda S Tanaka H Yamagata K Kim H. Non-clustered protocadherin. Cell adhesion migration (2011) 5:97–105. doi: 10.4161/cam.5.2.14374 21173574 PMC3084973

[7] Chen Y Xiang H Zhang Y Wang J Yu G. Loss of PCDH9 is associated with the differentiation of tumor cells and metastasis and predicts poor survival in gastric cancer. Clin Exp metastasis (2015) 32:417–28. doi: 10.1007/s10585-015-9712-7 25869928

[8] Lv J Zhu P Zhang X Zhang L Chen X Lu F. PCDH9 acts as a tumor suppressor inducing tumor cell arrest at G0/G1 phase and is frequently methylated in hepatocellular carcinoma. Mol Med Rep (2017) 16:4475–82. doi: 10.3892/mmr.2017.7193 PMC5647006 28791409

[9] Shi C Yang Y Zhang L Yu J Qin S Xu H. MiR-200a-3p promoted the malignant behaviors of ovarian cancer cells through regulating PCDH9. OncoTargets (2019) 12:8329–38. doi: 10.2147/ott.S220339 PMC6790212 31632082

[10] Wang C Chen Q Li S Li S Zhao Z Gao H. Dual inhibition of PCDH9 expression by miR-215-5p up-regulation in gliomas. Oncotarget (2017) 8:10287–97. doi: 10.18632/oncotarget.14396 PMC5354659 28055966

[11] Zhu P Lv J Yang Z Guo L Zhang L Li M. Protocadherin 9 inhibits epithelial-mesenchymal transition and cell migration through activating GSK-3β in hepatocellular carcinoma. Biochem Biophys Res Commun (2014) 452:567–74. doi: 10.1016/j.bbrc.2014.08.101 25172662

[12] Wang C Yu G Liu J Wang J Zhang Y Zhang X. Downregulation of PCDH9 predicts prognosis for patients with glioma. J Clin Neurosci (2012) 19:541–5. doi: 10.1016/j.jocn.2011.04.047 22300792

[13] Lionarons DA Hancock DC Rana S East P Moore C Murillo MM. RAC1^P29S^ induces a mesenchymal phenotypic switch via serum response factor to promote melanoma development and therapy resistance. Cancer Cell (2019) 36:68–83.e9. doi: 10.1016/j.ccell.2019.05.015 31257073 PMC6617390

[14] Uribe-Alvarez C Guerrero-Rodríguez SL Rhodes J Cannon A Chernoff J Araiza-Olivera D. Targeting effector pathways in RAC1^P29S^-driven malignant melanoma. Small GTPases. (2021) 12:273–81. doi: 10.1080/21541248.2020.1728469 PMC8205048 32043900

[15] Dalton LE Kamarashev J Barinaga-Rementeria Ramirez I White G Malliri A Hurlstone A. Constitutive RAC activation is not sufficient to initiate melanocyte neoplasia but accelerates malignant progression. J Invest Dermatol (2013) 133:1572–81. doi: 10.1038/jid.2013.23 23337888

[16] Kwei KA Finch JS Ranger-Moore J Bowden GT. The role of Rac1 in maintaining malignant phenotype of mouse skin tumor cells. Cancer Lett (2006) 231:326–38. doi: 10.1016/j.canlet.2005.02.031 15893875

[17] Vega FM Ridley AJ. Rho GTPases in cancer cell biology. FEBS Lett (2008) 582:2093–101. doi: 10.1016/j.febslet.2008.04.039 18460342

[18] Davis MJ Ha BH Holman EC Halaban R Schlessinger J Boggon TJ. RAC1P29S is a spontaneously activating cancer-associated GTPase. Proc Natl Acad Sci (2013) 110:912–7. doi: 10.1073/pnas.1220895110 PMC3549122 23284172

[19] Ellenbroek SI Collard JG. Rho GTPases: functions and association with cancer. Clin Exp Metastasis (2007) 24:657–72. doi: 10.1007/s10585-007-9119-1 18000759

[20] Bae YS Oh H Rhee SG Yoo YD. Regulation of reactive oxygen species generation in cell signaling. Molecules (2011) 32:491–509. doi: 10.1007/s10059-011-0276-3 PMC3887685 22207195

[21] Wu WS. The signaling mechanism of ROS in tumor progression. Cancer metastasis Rev (2006) 25:695–705. doi: 10.1007/s10555-006-9037-8 17160708

[22] Bedard K Krause KH. The NOX family of ROS-generating NADPH oxidases: physiology and pathophysiology. Physiol Rev (2007) 87:245–313. doi: 10.1152/physrev.00044.2005 17237347

[23] Harfouche R Malak NA Brandes RP Karsan A Irani K Hussain SN. Roles of reactive oxygen species in angiopoietin-1/tie-2 receptor signaling. FASEB J (2005) 19:1728–30. doi: 10.1096/fj.04-3621fje 16049136

[24] Ushio-Fukai M Alexander RW. Reactive oxygen species as mediators of angiogenesis signaling: role of NAD(P)H oxidase. Mol Cell Biochem (2004) 264:85–97. doi: 10.1023/b:mcbi.0000044378.09409.b5 15544038

[25] Binker MG Binker-Cosen AA Richards D Oliver B Cosen-Binker LI. EGF promotes invasion by PANC-1 cells through Rac1/ROS-dependent secretion and activation of MMP-2. Biochem Biophys Res Commun (2009) 379:445–50. doi: 10.1016/j.bbrc.2008.12.080 19116140

[26] Steinbrenner H Ramos MC Stuhlmann D Mitic D Sies H Brenneisen P. Tumor promoter TPA stimulates MMP-9 secretion from human keratinocytes by activation of superoxide-producing NADPH oxidase. Free Radical Res (2005) 39:245–53. doi: 10.1080/10715760500053487 15788229

[27] Overall CM López-Otín C. Strategies for MMP inhibition in cancer: innovations for the post-trial era. Nat Rev Cancer (2002) 2:657–72. doi: 10.1038/nrc884 12209155

[28] Marusak C Bayles I Ma J Gooyit M Gao M Chang M. The thiirane-based selective MT1-MMP/MMP2 inhibitor ND-322 reduces melanoma tumor growth and delays metastatic dissemination. Pharmacol Res (2016) 113:515–20. doi: 10.1016/j.phrs.2016.09.033 PMC5107128 27687955

[29] Bianchini F D'Alessio S Fibbi G Del Rosso M Calorini L. Cytokine-dependent invasiveness in B16 murine melanoma cells: role of uPA system and MMP-9. Oncol Rep (2006) 15:709–14. doi: 10.3892/or.15.3.709 16465434

[30] Tang ZY Liu Y Liu LX Ding XY Zhang H Fang LQ. RNAi-mediated MMP-9 silencing inhibits mouse melanoma cell invasion and migration in vitro and in vivo. Cell Biol Int (2013) 37:849–54. doi: 10.1002/cbin.10107 23554050

[31] Corsi JM Rouer E Girault JA Enslen H. Organization and post-transcriptional processing of focal adhesion kinase gene. BMC Genomics (2006) 7:198. doi: 10.1186/1471-2164-7-198 16889663 PMC1570463

[32] Sieg DJ Ilić D Jones KC Damsky CH Hunter T Schlaepfer DD. Pyk2 and src-family protein-tyrosine kinases compensate for the loss of FAK in fibronectin-stimulated signaling events but Pyk2 does not fully function to enhance FAK- cell migration. EMBO J (1998) 17:5933–47. doi: 10.1093/emboj/17.20.5933 PMC1170921 9774338

[33] Weis SM Lim ST Lutu-Fuga KM Barnes LA Chen XL Göthert JR. Compensatory role for Pyk2 during angiogenesis in adult mice lacking endothelial cell FAK. J Cell Biol (2008) 181:43–50. doi: 10.1083/jcb.200710038 18391070 PMC2287283

[34] Naser R Aldehaiman A Díaz-Galicia E Arold ST. Endogenous control mechanisms of FAK and PYK2 and their relevance to cancer development. Cancers (2018) 10:196. doi: 10.3390/cancers10060196 PMC6025627 29891810

[35] Yoon H Dehart JP Murphy JM Lim ST. Understanding the roles of FAK in cancer: inhibitors, genetic models, and new insights. J Histochem Cytochem (2015) 63:114–28. doi: 10.1369/0022155414561498 PMC4305513 25380750

[36] Zhu X Bao Y Guo Y Yang W. Proline-rich protein tyrosine kinase 2 in inflammation and cancer. Cancers (2018) 10:139. doi: 10.3390/cancers10050139 PMC5977112 29738483

[37] Sauter ER Yeo UC von Stemm A Zhu W Litwin S Tichansky DS. Cyclin D1 is a candidate oncogene in cutaneous melanoma. Cancer Res (2002) 62:3200–6. 12036934

[38] Kim JK Diehl JA. Nuclear cyclin D1: an oncogenic driver in human cancer. J Cell Physiol (2009) 220:292–6. doi: 10.1002/jcp.21791 PMC2874239 19415697

[39] Li Y Zheng Q Bao C Li S Guo W Zhao J. Circular RNA is enriched and stable in exosomes: a promising biomarker for cancer diagnosis. Cell Res (2015) 25:981–4. doi: 10.1038/cr.2015.82 PMC4528056 26138677

[40] Rong D Sun H Li Z Liu S Dong C Fu K. An emerging function of circRNA-miRNAs-mRNA axis in human diseases. Oncotarget (2017) 8:73271–81. doi: 10.18632/oncotarget.19154 PMC5641211 29069868

[41] Luan W Shi Y Zhou Z Xia Y Wang J. circRNA_0084043 promote malignant melanoma progression via miR-153-3p/Snail axis. Biochem Biophys Res Commun (2018) 502:22–9. doi: 10.1016/j.bbrc.2018.05.114 29777697

[42] Livak KJ Schmittgen TD. Analysis of relative gene expression data using real-time quantitative PCR and the 2(-delta delta C(T)) method. Methods (2001) 25:402–8. doi: 10.1006/meth.2001.1262 11846609

[43] Xie Z Zhou F Yang Y Li L Lei Y Lin X. Lnc-PCDH9-13:1 is a hypersensitive and specific biomarker for early hepatocellular carcinoma. EBioMedicine (2018) 33:57–67. doi: 10.1016/j.ebiom.2018.06.026 30045829 PMC6085584

[44] Daugaard M Nitsch R Razaghi B McDonald L Jarrar A Torrino S. Hace1 controls ROS generation of vertebrate Rac1-dependent NADPH oxidase complexes. Nat Commun (2013) 4:2180. doi: 10.1038/ncomms3180 23864022 PMC3759041

[45] Solé C Tramonti D Schramm M Goicoechea I Armesto M Hernandez LI. The circulating transcriptome as a source of biomarkers for melanoma. Cancers (2019) 11:70. doi: 10.3390/cancers11010070 PMC6356785 30634628

[46] Chen Z Chen J Wa Q He M Wang X Zhou J. Knockdown of circ_0084043 suppresses the development of human melanoma cells through miR-429/tribbles homolog 2 axis and wnt/β-catenin pathway. Life Sci (2020) 243:117323. doi: 10.1016/j.lfs.2020.117323 31954160

[47] Yang K Oak ASW Slominski RM Brożyna AA Slominski AT. Current molecular markers of melanoma and treatment targets. Int J Mol Sci (2020) 21:3535. doi: 10.3390/ijms21103535 PMC7278971 32429485

[48] Wang C Tao B Li S Li B Wang X Hu G. Characterizing the role of PCDH9 in the regulation of glioma cell apoptosis and invasion. J Mol Neurosci (2014) 52:250–60. doi: 10.1007/s12031-013-0133-2 24214103

[49] Chong SJF Lai JXH Qu J Hirpara J Kang J Swaminathan K. A feedforward relationship between active Rac1 and phosphorylated bcl-2 is critical for sustaining bcl-2 phosphorylation and promoting cancer progression. Cancer Lett (2019) 457:151–67. doi: 10.1016/j.canlet.2019.05.009 31103719

[50] Hinterleitner C Huelsenbeck J Henninger C Wartlick F Schorr A Kaina B. Rac1 signaling protects monocytic AML cells expressing the MLL-AF9 oncogene from caspase-mediated apoptotic death. Apoptosis (2013) 18:963–79. doi: 10.1007/s10495-013-0842-6 23624644

[51] Lee SC Sim N Clement MV Yadav SK Pervaiz S. Dominant negative Rac1 attenuates paclitaxel-induced apoptosis in human melanoma cells through upregulation of heat shock protein 27: a functional proteomic analysis. Proteomics (2007) 7:4112–22. doi: 10.1002/pmic.200700386 17952876

[52] Ren S Wei GH Liu D Wang L Hou Y Zhu S. Whole-genome and transcriptome sequencing of prostate cancer identify new genetic alterations driving disease progression. Eur Urol (2018) 73:322–39. doi: 10.1016/j.eururo.2017.08.027 28927585

[53] Wu S Tang Y Liu W. Circ_0084043 promotes cell proliferation and glycolysis but blocks cell apoptosis in melanoma via circ_0084043-miR-31-KLF3 axis. Open Life Sci (2020) 15:774–86. doi: 10.1515/biol-2020-0071 33817265 PMC7747509

[54] Tong H Zhao K Wang J Xu H Xiao J. CircZNF609/miR-134-5p/BTG-2 axis regulates proliferation and migration of glioma cell. J Pharm Pharmacol (2020) 72:68–75. doi: 10.1111/jphp.13188 31721211

[55] Rotte A Martinka M Li G. MMP2 expression is a prognostic marker for primary melanoma patients. Cell Oncol (2012) 35:207–16. doi: 10.1007/s13402-012-0080-x PMC13001128 22669775

[56] Li L Fan P Chou H Li J Wang K Li H. Herbacetin suppressed MMP9 mediated angiogenesis of malignant melanoma through blocking EGFR-ERK/AKT signaling pathway. Biochimie (2019) 162:198–207. doi: 10.1016/j.biochi.2019.05.003 31075281

[57] Ramirez JA Guitart J Rao MS Diaz LK. Cyclin D1 expression in melanocytic lesions of the skin. Ann Diagn Pathol (2005) 9:185–8. doi: 10.1016/j.anndiagpath.2005.04.018 16084449

[58] Oba J Nakahara T Abe T Hagihara A Moroi Y Furue M. Expression of c-kit, p-ERK and cyclin D1 in malignant melanoma: an immunohistochemical study and analysis of prognostic value. J Dermatol Sci (2011) 62:116–23. doi: 10.1016/j.jdermsci.2011.02.011 21454057

[59] George E Polissar NL Wick M. Immunohistochemical evaluation of p16INK4A, e-cadherin, and cyclin D1 expression in melanoma and Spitz tumors. Am J Clin Pathol (2010) 133:370–9. doi: 10.1309/AJCP52YVYCTLUOPI 20154275

[60] Eferl R Wagner EF. AP-1: a double-edged sword in tumorigenesis. Nat Rev Cancer (2003) 3:859–68. doi: 10.1038/nrc1209 14668816

[61] Jochum W Passegué E Wagner EF. AP-1 in mouse development and tumorigenesis. Oncogene (2001) 20:2401–12. doi: 10.1038/sj.onc.1204389 11402336

[62] Weiss C Bohmann D. Deregulated repression of c-jun provides a potential link to its role in tumorigenesis. Cell Cycle (2004) 3:111–3. doi: 10.4161/cc.3.2.648 14712066

[63] Chadee DN Kyriakis JM. Activation of SAPK/JNKs in vitro. Methods Mol Biol (2010) 661:59–73. doi: 10.1007/978-1-60761-795-2_3 20811976

[64] Feehan RP Shantz LM. Molecular signaling cascades involved in nonmelanoma skin carcinogenesis. Biochem J (2016) 473:2973–94. doi: 10.1042/BCJ20160471 PMC5198714 27679857

[65] Shaulian E Karin M. AP-1 in cell proliferation and survival. Oncogene (2001) 20:2390–400. doi: 10.1038/sj.onc.1204383 11402335

[66] Kappelmann M Kuphal S Meister G Vardimon L Bosserhoff AK. MicroRNA miR-125b controls melanoma progression by direct regulation of c-jun protein expression. Oncogene (2013) 32:2984–91. doi: 10.1038/onc.2012.307 22797068

[67] Wong CH Iskandar KB Yadav SK Hirpara JL Loh T Pervaiz S. Simultaneous induction of non-canonical autophagy and apoptosis in cancer cells by ROS-dependent ERK and JNK activation. PloS One (2010) 5:e9996. doi: 10.1371/journal.pone.0009996 20368806 PMC2848860

[68] Gaillard P Jeanclaude-Etter I Ardissone V Arkinstall S Cambet Y Camps M. Design and synthesis of the first generation of novel potent, selective, and in vivo active (benzothiazol-2-yl)acetonitrile inhibitors of the c-jun n-terminal kinase. J medicinal Chem (2005) 48:4596–607. doi: 10.1021/jm0310986 15999997

[69] Larkin J Minor D D'Angelo S Neyns B Smylie M Miller WH Jr. Overall survival in patients with advanced melanoma who received nivolumab versus investigator's choice chemotherapy in CheckMate 037: A randomized, controlled, open-label phase III trial. J Clin Oncol (2018) 36:383–90. doi: 10.1200/jco.2016.71.8023 PMC6804912 28671856

[70] Reck M Rodríguez-Abreu D Robinson AG Hui R Csőszi T Fülöp A. Pembrolizumab versus chemotherapy for PD-L1-Positive non-Small-Cell lung cancer. New Engl J Med (2016) 375:1823–33. doi: 10.1056/NEJMoa1606774 27718847

[71] Rittmeyer A Barlesi F Waterkamp D Park K Ciardiello F von Pawel J. Atezolizumab versus docetaxel in patients with previously treated non-small-cell lung cancer (OAK): a phase 3, open-label, multicentre randomised controlled trial. Lancet (2017) 389:255–65. doi: 10.1016/s0140-6736(16)32517-x PMC6886121 27979383

[72] Bellmunt J de Wit R Vaughn DJ Fradet Y Lee JL Fong L. Pembrolizumab as second-line therapy for advanced urothelial carcinoma. New Engl J Med (2017) 376:1015–26. doi: 10.1056/NEJMoa1613683 PMC5635424 28212060

[73] Powles T Durán I van der Heijden MS Loriot Y Vogelzang NJ De Giorgi U. Atezolizumab versus chemotherapy in patients with platinum-treated locally advanced or metastatic urothelial carcinoma (IMvigor211): a multicentre, open-label, phase 3 randomised controlled trial. Lancet (2018) 391:748–57. doi: 10.1016/s0140-6736(17)33297-x 29268948

[74] Ribas A Lawrence D Atkinson V Agarwal S Miller WH Jr Carlino MS. Combined BRAF and MEK inhibition with PD-1 blockade immunotherapy in BRAF-mutant melanoma. Nat Med (2019) 25:936–40. doi: 10.1038/s41591-019-0476-5 PMC8562134 31171879

