# Supplementary material for: Circ_0084043-miR-134-5p axis regulates PCDH9 to suppress melanoma
Source: Front Oncol. 2022 Oct 25;12:891476. doi: 10.3389/fonc.2022.891476 (PMC9641620; doi:10.3389/fonc.2022.891476)
Supplement: Supplementary file 1 [file Table_1.docx]

**Table S1.** The primers for selected genes and amplicon sizes.

| Genes | sequence( 5′→ 3′) | Product length |
| --- | --- | --- |
| PCDH9 | F: TCCCAACTCTGATGGGCCTTTGGG  R: GGCTCTGGTCAGGGTGTGCC | 217 bp |
| Rac1 | F: ACAAGCCGATTGCCGATGTGTTC  R: TGCCGCACCTCAGGATACCAC | 97 bp |
| Cyclin D1 | F:AGGAGAACAACTCTGACAACCACAATC  R:GCTCTTGATCGTCCTCTGACCAATAC | 93 bp |
| Pyk2 | F:GAGACCTACCGCTGTGAACTCATTC  R:CCTGATGGACCTGATCTGCTTGAAC | 141 bp |
| FAK | F:CTTCAAGAAGCCTTAACAATGCGTCAG  R:CACAGCTCCATGATTATCCAGACAGG | 101bp |
| MMP2 | F: TTTGACGGTAAGGACGGACTC  R: TACTCCCCATCGGCGTTC | 146 bp |
| MMP9 | F: CGAACTTTGACAGCGACAAGA  R: TCAGGGCGAGGACCATAGAG | 214 bp |
| GAPDH | F: TAAAAGCAGCCCTGGTGACC  R: CCACATCGCTCAGACACCAT | 88bp |
| PCDH9 | F: TCCCAACTCTGATGGGCCTTTGGG  R: GGCTCTGGTCAGGGTGTGCC | 217 bp |
| circ_0084043 | F: TTTCTCCAGTGACACCTCCCAGAG  R: GGGAACTGCTGAGGTTGCTTGG | 91 bp |

F：Forward Sequence; R: Reverse Sequence
