# Supplementary figures and images for: Circ_0084043-miR-134-5p axis regulates PCDH9 to suppress melanoma
Source: Front Oncol. 2022 Oct 25;12:891476. doi: 10.3389/fonc.2022.891476 (PMC9641620; doi:10.3389/fonc.2022.891476)

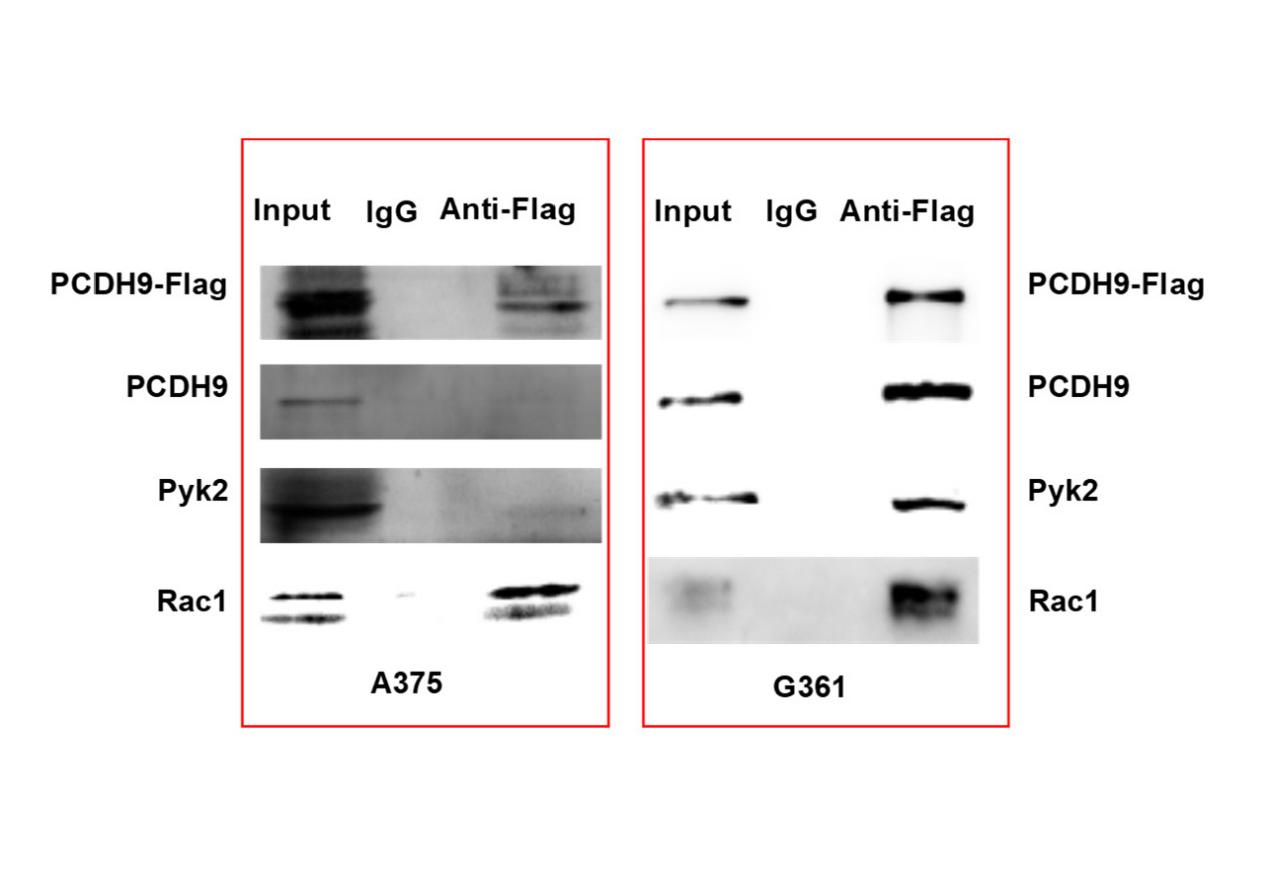

Supplement: Supplementary file 2 [file Image_1.jpeg]

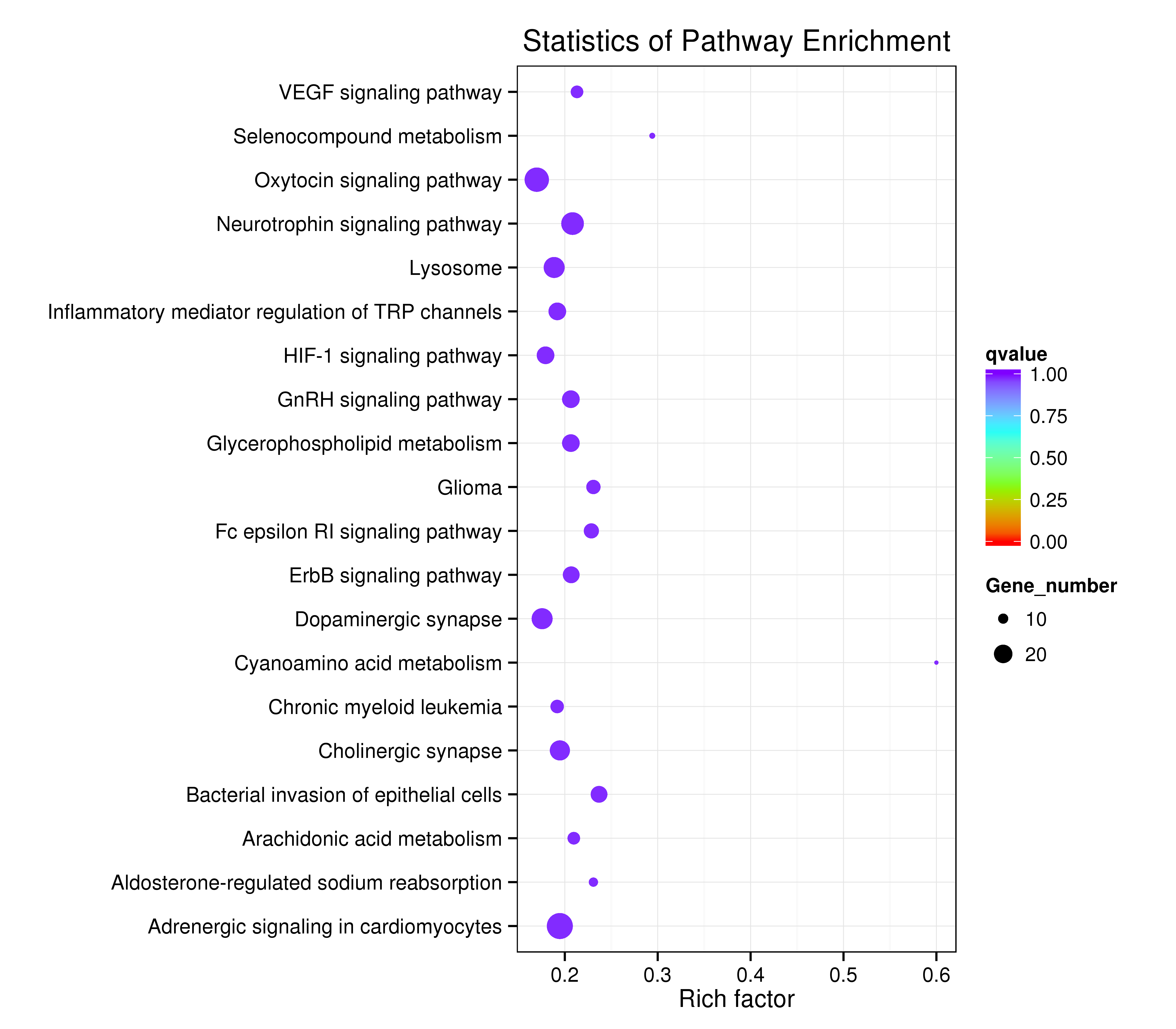

Supplement: Supplementary file 3 [file Image_2.png]

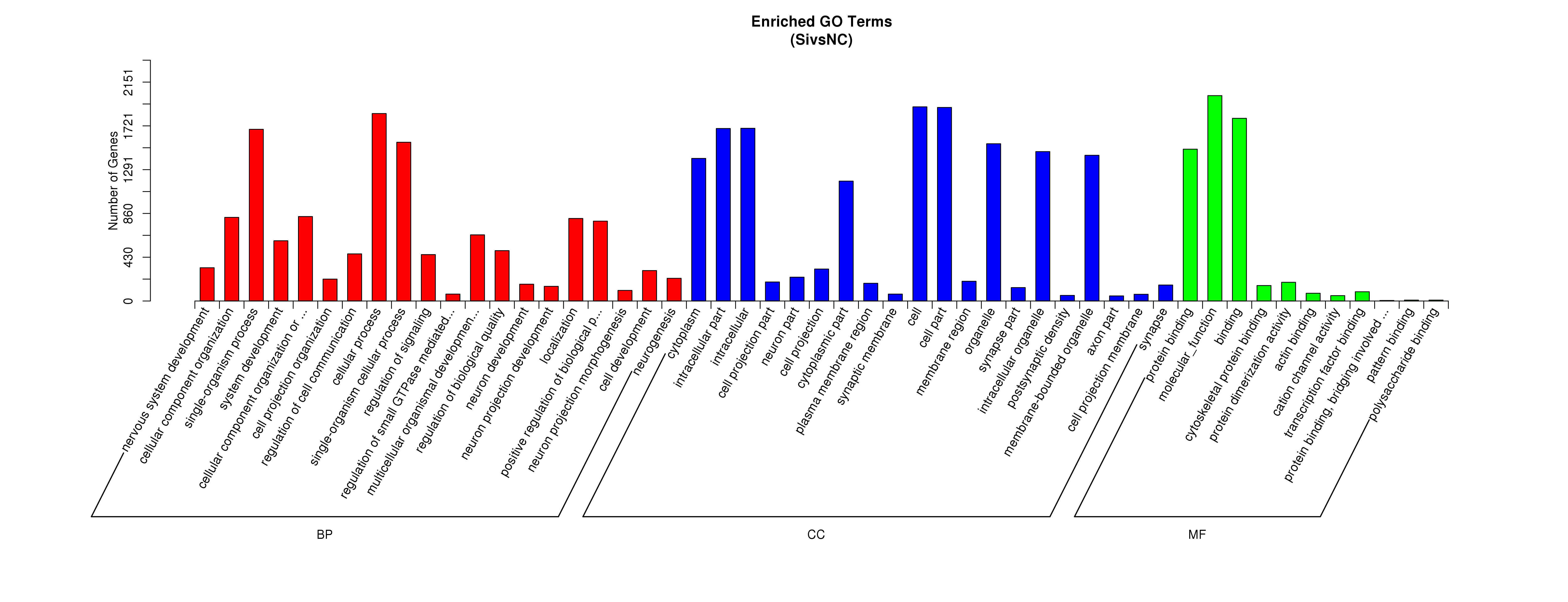

Supplement: Supplementary file 4 [file Image_3.png]
